# Data discovery with DATS: exemplar adoptions and lessons learned

**DOI:** 10.1093/jamia/ocx119

**Published:** 2017-12-08

**Authors:** Alejandra N Gonzalez-Beltran, John Campbell, Patrick Dunn, Diana Guijarro, Sanda Ionescu, Hyeoneui Kim, Jared Lyle, Jeffrey Wiser, Susanna-Assunta Sansone, Philippe Rocca-Serra

**Affiliations:** 1 Oxford e-Research Centre, Engineering Science, University of Oxford, Oxford, UK; 2 Northrop Grumman Information Systems Health IT, Rockville, MD, USA; 3 Health System Department of Biomedical Informatics, University of California, San Diego, La Jolla, CA, USA; 4 Inter-university Consortium for Political and Social Research, University of Michigan, Ann Arbor, MI, USA

**Keywords:** data discovery, data model, search engine, metadata

## Abstract

The DAta Tag Suite (DATS) is a model supporting dataset description, indexing, and discovery. It is available as an annotated serialization with schema.org, a vocabulary used by major search engines, thus making the datasets discoverable on the web. DATS underlies DataMed, the National Institutes of Health Big Data to Knowledge Data Discovery Index prototype, which aims to provide a “PubMed for datasets.” The experience gained while indexing a heterogeneous range of >60 repositories in DataMed helped in evaluating DATS’s entities, attributes, and scope. In this work, 3 additional exemplary and diverse data sources were mapped to DATS by their representatives or experts, offering a deep scan of DATS fitness against a new set of existing data. The procedure, including feedback from users and implementers, resulted in DATS implementation guidelines and best practices, and identification of a path for evolving and optimizing the model. Finally, the work exposed additional needs when defining datasets for indexing, especially in the context of clinical and observational information.

## INTRODUCTION

Discovery and access of datasets is crucial for propelling scientific discoveries. The DAta Tag Suite (DATS)^1^ model facilitates this and powers the DataMed data discovery index prototype^2^^,^^3^ developed by the National Institutes of Health (NIH) Big Data to Knowledge Initiative bioCADDIE project.^4^ DataMed aims to support dataset search as a counterpart of the longstanding and successful PubMed project for literature search.^5^ DATS’s role in the exchange of dataset information is expected to be similar to the Journal Article Tag Suite model^6^ used for metadata exchange between publishers and PubMed. DATS’s development^1^^,^^7^ has been ongoing for over 2 years and incorporates feedback from a variety of data modelers, developers, and organizations associated with research data. At present, the NIH National Library of Medicine is exploring DATS and its possible role in ongoing efforts to make a broader range of biomedical data more readily discoverable.

When referring to the principles for FAIR data,^8^ DATS’s focus is on datasets’ *findability* and *accessibility*, leaving detailed descriptions enabling *interoperability* and *reuse* to the original data sources. Thus, DATS was designed to expose the key elements for data discovery through a single interface**,** where data are then accessed at the source. DATS is annotated with the schema.org vocabulary, used by major search engines, and thus supports making the datasets visible and discoverable on the wider web.

At the time of writing (August 2017), DataMed had indexed 74 data sources with >2.3 million datasets covering an extensive set of subjects and data types. The ingestion process was carried out mainly by DataMed curators and developers by mapping the schemas of data sources to DATS and consulting with the data sources’ developers when relevant. In order to scale DataMed, data repositories will need to provide their metadata in DATS format, as journals provide Journal Article Tag Suite metadata for their articles to be indexed in PubMed. DATS^1^ has a set of generic elements, applicable to any dataset, and an extended set of elements for specialized data types, especially in the biomedical domain. The properties for each element are identified and defined. DATS is represented with JavaScript Object Notation (JSON) schemas, and documentation and validation code are provided to check dataset representation against the schemas.^9^

Here we present the novel processes and feedback after experts on 3 very different data sources performed mappings to DATS in order to ingest their datasets into DataMed, as follows:

- Clinical and molecular datasets in immunology from the Immunology Database and Analysis Portal (ImmPort),
- Social and behavioral sciences from the Inter-university Consortium for Political and Social Research (ICPSR), and
- Health care data as represented in the Observational Medical Outcomes Partnership (OMOP) Common Data Model (CDM).

We explore the question of how feasible and complex it is to support mapping from a data resource model to DATS, and discuss the challenges, lessons learned, and next steps in the evolution of DATS and its documentation to facilitate the ingestion process of new data sources into DataMed in a scalable way.

## DATS IMPLEMENTATIONS

### ImmPort

The ImmPort project^10^^,^^11^ offers advanced information technology for archiving, exchanging, and disseminating clinical and molecular datasets produced by research teams funded primarily (though not exclusively) by the NIH National Institute of Allergy and Infectious Diseases Division of Allergy, Immunology, and Transplantation.^12^

At the core of ImmPort is a data warehouse holding metadata about clinical and mechanistic results. The study summary metadata, such as the purpose, endpoints, and associated publications, share features with the ISA metadata model.^13^ Molecular phenotyping drives the capture of metadata for specific techniques (eg, flow cytometry and polymerase chain reaction), which are annotated with ontological terms.^14^ Study cohorts or arms are described reusing elements of the Clinical Data Interchange Standards Consortium Study Data Tabulation Model.^15^ Standard demographic attributes and terms are used for NIH study subjects, and samples are described with anatomical ontologies. ImmPort provides open access data for registered users and registration is free, as well as support for holding private data.

#### ImmPort to DATS mapping

The ImmPort data model^16^^,^^17^ was compared with the DATS specification.^7^^,^^17^ While DATS is dataset-centric, ImmPort is a study/experiment-based model whose primary object is a study^18^^,^^19^ that can have multiple experiments/lab tests/assessments. The alternatives were to map dataset to study or dataset to experiment. As most of ImmPort’s metadata (eg, arms, publications, personnel, subjects, clinical data) is linked to studies, an ImmPort dataset was defined as the data produced by a single study. The alternative option would result in significant redundancy (as the experiments’ high-level information is shared at the study level).

A subset of the ImmPort relational model was extracted programmatically, transformed to the DATS instance specification using templates, and then validated against the DATS schemas.^20^

### ICPSR

ICPSR^21^ is a disciplinary data repository focused on social and behavioral sciences research. For >50 years, it has been archiving, curating, and providing access to an extensive collection in excess of 10 000 studies and almost 5 million variables. ICPSR partners with several United States federal agencies and foundations, including NIH, and thus it holds a significant amount of health measures or outcomes. ICPSR shares its metadata widely, including creating machine-readable metadata using schema.org markup in JSON-LD format^22^ and enabling bulk metadata exports.^23^ As the traditional user is from the social or behavioral sciences, mapping ICPSR’s metadata into DATS and broadening their reach through DataMed increases findability and discoverability, especially for the biomedical community.

#### ICPSR to DATS mapping

All ICPSR collection-level metadata rely on the Data Documentation Initiative (DDI) metadata standard.^19–26^ The study-level metadata served as the starting point for mapping to DATS. While DDI includes 1154 possible elements (objects and attributes), the core fields mapped consisted of roughly 12, shown in Table 1.

**Table 1.** Mapping of 12 ICPSR key metadata fields to DATS descriptor core elements

ICPSR field	DATS dataset entity attributes
Study number	identifierInformation
Study title/dataset title	Title
Summary	Description
Kind of data/data type	DataType
Distributor	StoredIn
Terms of use	License
Download URL	doi
Investigator	Creator
Time period, collection date, release date, date updated	date_info
Version	Version
File size	Size

### OMOP CDM

OMOP CDM^27^^,^^28^ provides a means to transform heterogeneous formats of observational health data into a common form, enabling integrative systematic analysis.^27^ It is maintained by the Observational Health Data Sciences and Informatics (OHDSI, pronounced “odyssey”) program.^27^ It was originally developed to support studies investigating the effectiveness of medical products,^27^ and therefore shares many features with the Clinical Data Interchange Standards Consortium Study Data Tabulation Model.^13^ However, its genericity means it is also suited to accommodate electronic health records and capture patient-related information independent of whether a person is enrolled in a study or not. OMOP is now widely used to support various types of health care studies that require health care data such as claims, administrative, and clinical data.^27^ Therefore, an OMOP archive does not need to declare a project or a study. However, it may be used to define and declare cohorts, ie, groups of patients whose characteristics match a set of selection criteria as defined by clinicians.

#### OMOP to DATS mapping

Health care datasets are underrepresented in DataMed, primarily because of their limited availability due to patient privacy issues. However, we anticipate that health care datasets will become increasingly available for secondary analysis and reuse as a consequence of research programs such as the All-of-Us/Precision Medicine Initiative collecting and analyzing clinical, environmental, lifestyle, and biological data of patients to discover innovative ways to prevent and treat human diseases.^29–31^ Data access should be via appropriate training and formal access requests^27^ to ensure that even deidentified data are used appropriately, as they contain detailed information on the clinical care of patients.

As before, the first mapping challenge was to identify a dataset from the OMOP CDM perspective, which is patient-centric, resulting in 2 potential interpretations:

1. The whole model, and thus all the information about clinical encounters for all the patients.
2. A cohort, or group of patients according to a set of specified criteria, and the information of their clinical care.

For the purpose of this manuscript, we only considered the first interpretation and performed the mapping between OMOP CDM 5.1.0 and DATS 2.2. The mapping and an example DATS representation of a dataset by Observational Health Data Sciences and Informatics^28^ are available online.^9^

## DISCUSSION: LESSONS LEARNED AND NEXT STEPS

Each of the mapping efforts identified specific sets of issues. All point to the level of granularity necessary for an export to allow dataset discoverability while maintaining accuracy.

### How to identify a dataset and required extensions for DATS

Identifying what is a dataset for a particular source is crucial for setting up an indexing pipeline to DataMed. The source’s cardinal properties need to be matched against the dataset typology recognized by DATS:

- An entire collection of records (as in the OMOP CDM, where the dataset is the whole model).
- A subset of records (as for OMOP CDM cohorts).
- Individual records: the lowest level of granularity defined by the repository at which minting identifiers (or defining accession numbers) can be done. A distinction needs to be made between study-centric repositories (eg, ImmPort, ICPSR, GEO, MetaboLights) and knowledge bases (eg, UniProt) or observational patient health records (eg, OMOP CDM).

For OMOP CDM, to relate the dataset with the patient's information that composes it, a new association between dataset and material is needed. Also, providing summary data (eg, number of samples/patients) would be beneficial, especially for datasets with access restrictions.

### How to use DATS dimensions

**Figure 1.**
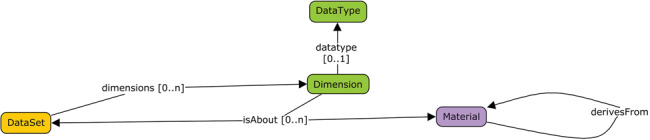
Section of the DATS model involving dimension

The second cluster of challenges related to using the DATS Dimension entity (see Figure 1), which is a high-level representation of quantitative or qualitative properties of an entity. All groups correctly associated the notion of “dimension” to that of “variable.”

ImmPort included as DATS Dimensions: (1) the measurement techniques used in experiments (eg, ELISA, ELISPOT, flow cytometry); (2) the reported names of collections of laboratory processes (eg, chemistry test, blood cell count); and (3) assessment panels or evaluations of subjects (eg, questionnaires, ratings based on a reference scale) that did not involve drawing samples from the subjects (eg, medical history, atopic dermatitis assessment). In the future, the ImmPort will also include other elements (eg, laboratory tests) as dimensions.

In the ICPSR case, data collections currently define >5 million variables. The DDI model offers a highly refined notion of variables, including names, values, labels, question text, and frequency listings. Furthermore, DDI details cases of variable derivation, summarization, and so on. DATS dimensions are not entirely analogous to DDI variables: they have a larger scope, while DDI variables are primarily intended for quantitative data. Still, most of the properties of a DATS dimension can be straightforwardly mapped to elements in the DDI variable description (see Table 2).

**Table 2.** Mapping between DATS dimension and DDI variable

DATS dimension	DDI variable
Identifier	ID
Name	Name
Types	interval/nature/format
DataType	Kind of data
PartOf	(Study) title
Description	Descriptive text/ question text
Values	Valid values range
Unit	Measurement unit
isAbout	Concept
extraProperties	Notes

Some additional DDI variable fields are not captured in DATS, but owing to its being lightweight and its focus on findability, DATS representation is expected to be streamlined and entail a loss of information.

In OMOP CDM, observation and measurement include both procedural and variable information. While observations are clinical facts obtained through examination or questioning, measurements are numerical or categorical values obtained through a systematic and standardized test. When mapping to DATS, these 2 entities were split between the DataAcquisition process information and the values acquired, represented as Dimension(s).

OMOP procedures were also initially mapped to DATS Dimensions. However, the recommended representation is to use “Treatment.” DATS Treatment should not be interpreted as being restricted to clinical interventions. It is meant for reporting any form of exposure in an interventional or observational context. Once this is clarified, mapping OMOP Exposures (device exposure, drug exposure, or procedure occurrence) is straightforward, even if the DATS is not as granular.

Another discussion was about use of possible missing features to better qualify, describe, and index information related to variables. Questions arose as to whether Dimension’s values are for the range of permissible values, for the range of actual values, or for some variable summarization such as {mix|max|median}. The latter would entail some data processing and therefore was not considered. The former boils down to defining a variable’s value range. This echoes discussions at a dedicated bioCADDIE workshop, where it was discussed whether common data elements and forms should be indexed in DataMed. This discussion and its impact on DATS are ongoing.

#### Documentation and additional support infrastructure

DATS documentation available to perform the mapping was the JSON schemas, instances, and validation code^18^; the diagrams and spreadsheets in the specification^19^; and the DATS manuscript^1^ and recorded presentations.

As future additions to the DATS documentation and support for DataMed ingestion, ImmPort suggested a test site where DATS instances could be loaded and validated against the specification. The frequency of incremental updates of ingested databases needs to specified, as well as the frequency of updates to the DATS specification. For the latter, we expect that this type of exercise will help in making sure that the relatively stable DATS core model will be suitable to support a broad range of datasets across domains.

### Next steps

The results of this mapping analysis will be reflected in the next DATS version, accompanied by its documentation and supporting tools.

## FUNDING

The bioCADDIE project is funded by grant U24AI117966 from the NIH National Institute of Allergy and Infectious Diseases, as part of the Big Data to Knowledge Initiative program. The DATS work in schema.org and related bioschemas by S-AS, AG-B, and PR-S is supported by the EU ELIXIR EXCELERATE project (H2020-INFRADEV-1-2015-1 676559) and ELIXIR-UK Node (UK BBSRC BB/L005069/1).

## COMPETING INTERESTS

The authors declare no competing interests.
